# *Hedysarum* L. (Fabaceae: Hedysareae) Is Not Monophyletic – Evidence from Phylogenetic Analyses Based on Five Nuclear and Five Plastid Sequences

**DOI:** 10.1371/journal.pone.0170596

**Published:** 2017-01-25

**Authors:** Pei-Liang Liu, Jun Wen, Lei Duan, Emine Arslan, Kuddisi Ertuğrul, Zhao-Yang Chang

**Affiliations:** 1 College of Life Sciences, Northwest A&F University, Yangling, Shaanxi, China; 2 Department of Botany, National Museum of Natural History, Smithsonian Institution, Washington D.C., United States of America; 3 Key Laboratory of Plant Resources Conservation and Sustainable Utilization, South China Botanical Garden, Chinese Academy of Sciences, Guangzhou, Guangdong, China; 4 Department of Biology, Faculty of Science, Selçuk University, Konya, Turkey; Chinese Academy of Medical Sciences and Peking Union Medical College, CHINA

## Abstract

The legume family (Fabaceae) exhibits a high level of species diversity and evolutionary success worldwide. Previous phylogenetic studies of the genus *Hedysarum* L. (Fabaceae: Hedysareae) showed that the nuclear and the plastid topologies might be incongruent, and the systematic position of the *Hedysarum* sect. *Stracheya* clade was uncertain. In this study, phylogenetic relationships of *Hedysarum* were investigated based on the nuclear ITS, ETS, *PGDH*, *SQD1*, *TRPT* and the plastid *psbA-trnH*, *trnC-petN*, *trnL-trnF*, *trnS-trnG*, *petN-psbM* sequences. Both nuclear and plastid data support two major lineages in *Hedysarum*: the *Hedysarum s*.*s*. clade and the *Sartoria* clade. In the nuclear tree, *Hedysarum* is biphyletic with the *Hedysarum s*.*s*. clade sister to the *Corethrodendron + Eversmannia + Greuteria + Onobrychis* clade (the CEGO clade), whereas the *Sartoria* clade is sister to the genus *Taverniera* DC. In the plastid tree, *Hedysarum* is monophyletic and sister to *Taverniera*. The incongruent position of the *Hedysarum s*.*s*. clade between the nuclear and plastid trees may be best explained by a chloroplast capture hypothesis via introgression. The *Hedysarum* sect. *Stracheya* clade is resolved as sister to the *H*. sect. *Hedysarum* clade in both nuclear and plastid trees, and our analyses support merging *Stracheya* into *Hedysarum*. Based on our new evidence from multiple sequences, *Hedysarum* is not monophyletic, and its generic delimitation needs to be reconsidered.

## Introduction

The legume family (Fabaceae) is the third largest flowering plant family with about 19,500 species in about 751 genera. The family exhibits a high level of species diversity and evolutionary success in various ecosystems worldwide [1]. The genus *Hedysarum* L. (Fabaceae: Hedysareae) consists of about 160 species of perennial herbs to rarely shrublets. It mainly distributes in temperate Eurasia, with a few species in North Africa and North America. Species of *Hedysarum* adapt to diverse habitats in temperate forests, steppes, alpine regions and the Tibetan plateau. This genus is generally characterized by brown membranous connate stipules, imparipinnate leaves, non-persistent corollas at fruit maturity, backward turning standard petal, right- to obtuse-angle-shaped keels and most prominently, lomented legumes with 2-several seeds [2, 3]. Some species of *Hedysarum* are good fodder plants, such as *H*. *dahuricum* Turcz. ex B.Fedtsch. and *H*. *petrovii* Yakovlev in arid regions, and *H*. *neglectum* Ledeb. in alpine regions [4, 5].

The generic delimitation of *Hedysarum* has been highly problematic. Established by Linnaeus [6], *Hedysarum* consisted of 54 species at his time [7]. However, *Hedysarum* sensu Linnaeus was unnatural with 51 species subsequently transferred to 16 other genera such as *Alhagi* Gagnebin, *Desmodium* Desv., *Onobrychis* Mill. and *Sulla* Medik. [2, 7]. With more species being discovered, Fedtschenko [8] divided *Hedysarum* into seven sections mainly based on their habits and morphology of stems and loments. Choi & Ohashi [2] revised Fedtschenko’s classification of *Hedysarum* based on their comprehensive morphological studies. They segregated the genera *Corethrodendron* Fischer & Basiner and *Sulla* from *Hedysarum*, and transferred the monotypic genus *Stracheya* Benth. [9] into *Hedysarum* as *H*. sect. *Stracheya* (Benth.) B.H.Choi & H.Ohashi. They also merged *H*. sect. *Multicaulia* (Boiss.) B.Fedtsch., *H*. sect. *Crinifera* (Boiss.) B.Fedtsch. and *H*. sect. *Subacaulia* (Boiss.) B.Fedtsch. into a broadly defined *H*. sect. *Multicaulia s*.*l*. [2].

Recent phylogenetic studies of Hedysareae [10, 11] did not support the monophyly of *Hedysarum* as circumscribed by Fedtschenko [8] nor by Choi & Ohashi [2]. Separations of the genera *Corethrodendron* and *Sulla* from *Hedysarum* were supported by molecular data [10, 11]. A new genus, *Greuteria* Amirahmadi & Kaz. Osaloo., was split from *Hedysarum* based on phylogenetic reconstruction [10, 11]. The monotypic *Sartoria* Boiss. & Heldr. was nested within *Hedysarum* sect. *Multicaulia s*.*l*. Therefore *Sartoria hedysaroides* Boiss. & Heldr. was transferred to *Hedysarum* as *H*. *anatolicum* Amirahmadi & Kaz. Osaloo [10]. After these treatments, *Hedysarum* was suggested to be monophyletic in the plastid trees [10, 11]. However, *Hedysarum* was not monophyletic in the nuclear ITS trees, and its relationship with other genera in Hedysareae was uncertain due to low support values of the ITS trees [10, 11].

With an extensive taxon sampling scheme and more molecular data, Duan et al. [11] recognized three main clades of *Hedysarum*: the *H*. sect. *Hedysarum* clade; the re-defined *H*. sect. *Multicaulia s*.*l*. clade excluding *H*. *kumaonense* Benth. ex Baker and *H*. *lehmannianum* Bunge, but including *Sartoria hedysaroides*; and the re-delimited *H*. sect. *Stracheya* clade consisting of *H*. *tibeticum* (Benth.) B.H.Choi & H.Ohashi, *H*. *kumaonense* and *H*. *lehmannianum*. The *H*. sect. *Stracheya* clade was sister to the *H*. sect. *Multicaulia s*.*l*. clade with very low support [11]. However, this relationship was not supported by Amirahmadi et al. [10]. The systematic position of the *H*. sect. *Stracheya* clade within *Hedysarum* was thus uncertain.

Previous phylogenetic hypotheses on *Hedysarum* and Hedysareae largely relied on plastid data since the nuclear ITS sequence showed limited resolution concerning the deep relationships [10, 11]. Sequencing more plastid genes may increase phylogenetic precision, however, accurate inference of the phylogenetic history of this group requires nuclear data [12, 13]. Nuclear genes are considered as an important complement or alternative to plastid ones due to their biparental inheritance, and are less vulnerable to hybridizations and introgressions than organelle genes [1, 14, 15].

To our knowledge, only the ribosomal internal transcribed spacer (ITS) sequence from the nuclear genome has been used to infer the phylogeny of *Hedysarum*. The ribosomal external transcribed spacer (ETS) has also been widely used in phylogenetic studies [13], and has an evolutionary rate at least as fast as the ITS sequence [16]. Furthermore, several studies have developed other nuclear sequences for phylogenetic reconstruction in various taxonomic groups of Fabaceae [12, 17, 18]. The *SQD1* (UDP sulfoquinovose synthase gene) belongs to the low copy conserved ortholog set (COS) genes [19]. It has been used in a phylogenetic study of the caesalpinioid legumes [18]. The nuclear coding *PGDH* (putative phosphogluconate dehydrogenase gene) and *TRPT* (putative triosephosphate translocator gene) sequences are exon-derived, putative orthologs, single copy genes [12], and were used in a phylogenetic study of the Hologalegina legumes [17].

In the present study, we employed the nuclear ITS, ETS, *PGDH*, *SQD1* and *TRPT* sequences and the plastid *psbA-trnH*, *trnC-petN*, *trnL-trnF*, *trnS-trnG*, and *petN-psbM* sequences to test (1) the monophyly of *Hedysarum*, and (2) the systematic position of the *Hedysarum* sect. *Stracheya* clade.

## Materials and Methods

### Ethics statement

The plant materials used in this study did not involve protected or endangered species. No specific permits were required for the collection of samples. Voucher information of all the samples was given in S1 Appendix.

### Taxon sampling

A total of 58 accessions were included in this study (S1 Appendix), representing all genera in the tribe Hedysareae and all sections and major infra-sectional clades in *Hedysarum* as recognized by Duan et al. [11]. *Hedysarum boveanum* Bunge ex Basiner, *H*. *denticulatum* Regel, *H*. *minjanense* Rech.f., *H*. *poncinsii* Franch., and *H*. *syriacum* Boiss. were placed in sect. *Multicaulia s*.*l*. [2] based on morphological characters [20–22], and they were sampled for the first time to test their phylogenetic positions. *Alhagi sparsifolia* Shap. ex Keller & Shap. was selected as the outgroup based on previous results [10,11]. Some of the ITS, *psbA-trnH*, and *trnL-trnF* sequences used in this study were published by Duan et al. [11] and Amirahmadi et al. [10], while all other sequences were generated by the present study (S1 Appendix). Voucher information of DNA sequences was listed in S1 Appendix.

### DNA extraction, PCR and sequencing

Total genomic DNAs were extracted from silica-gel dried leaf material or herbarium specimen using either the Plant DNA Extraction Kit AGP965/960 (AutoGen, Holliston, Massachusetts, USA) or the DNeasy Plant Mini Kit (Qiagen, Valencia, California, USA).

Primers used for amplification and sequencing were “ITS5a” and “ITS4” for ITS [23], “ETS-Hedy” (CCYTGWGCYRTTGTGCCTTGG, designed in this study) and “18S-IGS” [16] for ETS, forward and reverse primers for *SQD1* [19], forward and reverse primers for *PGDH* and *TRPT* [12], “psbAF” and “trnHR” for *psbA-trnH* intergenic region [24], “trnC” and “petN 1R” for *trnC-petN* intergenic region [25], “trnS” [26] and “5’trnG2S” [27] for *trnS-trnG* intergenic region, “ycf6F” and “psbMR” [27] for the *petN-psbM* intergenic region (the *ycf6* gene has been renamed as *petN*, see [28, 29]), “c” and “f” for *trnL* intron plus *trnL-trnF* intergenic region [30].

Polymerase chain reaction (PCR) was performed in a 25μl volume with the following components: 10× reaction buffer, 200μmol·L^-1^ of each dNTP, 10μg BSA, 0.4μmol·L^-1^ of each primer, 2.5mmol·L^-1^ MgCl_2_, 1U of BIOLASE DNA Polymerase (Bioline USA Inc., Taunton, Massachusetts), 1–7.5μL template DNA. The amplification conditions were 3 min at 95°C, followed by 36–40 cycles of 1 min at 94°C, 1 min at 50–60°C, and 1–1.5 min at 72°C, then a final extension at 72°C for 7–10 min. The PCR products were purified using ExoSAP-IT (USB Corporation, Cleveland, Ohio, USA) or the polyethylene glycol (PEG) precipitation procedure [31]. Cycle sequencing reactions were conducted in both directions using the amplification primers and the BigDye 3.1 reagents. After being cleaned up by the Sephadex columns, the sequencing products were run on an ABI 3730 automated sequencer (Applied Biosystems, Foster City, California, USA). Sequences were assembled using the program Geneious v.8.1.2 [32] (http://www.geneious.com/). All sequences have been deposited in GenBank and the accession numbers were listed in S1 Appendix.

### Phylogenetic analysis

Multiple sequence alignments were conducted in Geneious v.8.1.2 using MUSCLE [33] with default settings, followed by manual adjustments. For the non-coding sequences, ambiguously aligned regions were removed from the matrix prior to phylogenetic analysis. Insertions and deletions (indels) in the data matrices were coded as binary characters using the program SeqState [34] according to the “simple coding” method [35]. The binary characters were combined with the DNA data as the additional partition of the matrix. SequenceMatrix [36] was employed to assemble combined datasets.

### Single locus analysis

Phylogenetic reconstructions were first conducted by using single locus datasets. The best-fit nucleotide substitution model for each of the 10 individual sequences was determined using the Bayesian Information Criterion (BIC) in jModelTest v.2.1.7 [37]. For the ITS dataset, boundaries of the 5.8S, ITS1 and ITS2 regions were determined by comparing with the published 5.8S sequence of *Vicia faba* L. [38], and models for the ITS1, 5.8S and ITS2 regions were determined separately (see Table 1).

**Table 1:** Characteristics of individual datasets: alignment length, the number and percentage of constant, variable and potentially parsimony-informative (Pi) sites, the number of coded indel(s), and the best-fit nucleotide substitution model determined by BIC.

Dataset	Length	Constant (%)	Variable (%)	Pi (%)	Indel	Model
**ETS**	346	131 (37.9%)	215 (62.1%)	149 (43.1%)	45	HKY+G
**ITS1**	285	126 (44.2%)	159 (55.8%)	112 (39.3%)	55	TIM3ef+G
**5.8S**	165	152 (92.1%)	13 (7.9%)	6 (3.6%)	2	TPM3+I
**ITS2**	229	108 (47.2%)	121 (52.8%)	86 (37.6%)	22	TrNef+G
***PGDH***	405	304 (75.1%)	101 (24.9%)	67 (16.5%)	0	K80+I
***SQD1***	273	212 (77.7%)	61 (22.3%)	41 (15.0%)	0	TPM1+G
***TRPT***	330	215 (65.2%)	115 (34.8%)	65 (19.7%)	7	HKY+G
***psbA-trnH***	403	300 (74.4%)	103 (25.6%)	58 (14.4%)	31	TPM1uf+I+G
***trnC-petN***	1221	882 (72.2%)	339 (27.8%)	213 (17.4%)	110	TVM+G
***trnL-trnF***	1068	881 (82.5%)	187 (17.5%)	110 (10.3%)	60	TVM+G
***trnS-trnG***	692	485 (70.1%)	207 (29.9%)	142 (20.5%)	69	TPM1uf+G
***petN-psbM***	1316	999 (75.9%)	317 (24.1%)	198 (15.0%)	104	TVM+G

Bayesian inferences (BI) were conducted in MrBayes v.3.2.5 [39, 40]. When models (such as TIM3ef, TrNef, TPM1, TVM) determined by BIC were not directly available in MrBayes, we transformed a GTR model in MrBayes by fixing the six nucleotide substitution rates and four nucleotide state frequencies to the values as calculated by jModelTest for each dataset. The model applied to all the coded binary partitions was a default Standard Discrete Model in MrBayes [41]. For the ITS dataset, the partitions were done for ITS1, 5.8S and ITS2 separately. In the Bayesian inference, two independent analyses starting from different random trees with three heated and one cold chain were run for 10,000,000 generations, and trees were sampled every 1,000 generations (10,000 trees sampled in total). The first 2,500 trees (25%) were discarded as burn-in, and the remaining trees were used to construct a 50% majority-rule consensus tree and posterior probabilities (PP). Tree visualization was achieved in FigTree v1.4.3 (http://tree.bio.ed.ac.uk/software/figtree/).

Phylogenetic reconstructions were also carried out with maximum parsimony (MP) and maximum likelihood (ML) analyses using PAUP* 4.0b10 [42] and RAxML v.8.2 [43], respectively. The MP bootstrap analysis was performed with the following settings: heuristic search, TBR branch-swapping, 1,000 bootstrap replicates, random addition sequence with 10 replicates, a maximum of 1,000 trees saved per round. The ML rapid bootstrap analysis was conducted with a random seed, 1,000 alternative runs, and the same partition scheme as in the Bayesian analysis. Model parameters were estimated and optimized for each partition of the dataset by RAxML with the GTRCAT commands. Bootstrap support values from the MP (PBS) and ML (LBS) analyses were labeled on the corresponding branches of the BI trees.

### Concatenated data analysis

We conducted incongruence length difference (ILD) test [44] to assess whether different datasets could be concatenated for phylogenetic reconstruction. The ILD tests were performed in PAUP* 4.0b10 [42] by using only the informative characters [45] with heuristic search, 1,000 replicates, simple addition sequence (with a maximum of 1,000 trees saved per replicate), and tree-bisection-reconnection (TBR) branch-swapping algorithm. When the ILD test found a *p* value greater than 0.01, datasets were concatenated for phylogenetic analysis [46].

BI, MP and ML analyses were conducted for the concatenated datasets by using the same methods as in the single locus analyses. For BI and ML, the best partitioning scheme and substitution model were determined by using PartitionFinder v.1.1.1 [47] with the following settings: linked branch length, BIC metric for model and partitioning selection, and the greedy algorithm. Data blocks in PartitionFinder were set as the followings: the ETS, ITS1, 5.8S and ITS2 datasets, the first, second and third codon position of each of the *PGDH*, *SQD1* and *TRPT* datasets, and the *psbA-trnH*, *trnC-petN*, *trnL-trnF*, *trnS-trnG* and *petN-psbM* datasets.

We performed the approximately unbiased (AU) tests [48] using CONSEL v.0.2 [49] to test statistical support of incongruence between nuclear and plastid topologies. Topological constraints were generated from the ML trees using MEGA v.6.0 [50]. Per site log likelihoods for each topology were estimated in RAxML v.8.2 [43] under the GTR + G model. The dataset partition scheme was the same as in the ML analysis.

### Coalescent analysis

The Bayesian coalescent-based analyses for multi-locus data were performed using *BEAST [51] as implemented in the BEAST v.2.4.3 package [52]. Two sets of analyses were conducted: one with all five nuclear sequences, another with all five plastid sequences. The substitution model for each sequence was that used in the single locus analysis, and model parameters were unlinked among partitions. The lognormal relaxed clock model, the linear with constant root population function and the Yule speciation model were used. Four independent runs of 100,000,000 and 500,000,000 generations were performed for the nuclear data and the plastid data, respectively. Samples were stored every 5,000 generations. Tracer v.1.6 (http://beast.bio.ed.ac.uk/Tracer) was used to check the effective sample sizes (ESSs) of the parameters and the convergence of different runs. After removing the 10% burn-in of each run, the log and tree files from different runs were combined by using LogCombiner [52]. In the combined results, the ESSs of the sampled parameters all exceeded 200. The maximum clade credibility tree was annotated using TreeAnnotator [52].

### Bayesian concordance analysis

We carried out the Bayesian Concordance Analysis (BCA) [53] for the five nuclear loci as well as the five plastid loci using the BUCKy v.1.1.4 package [54]. Gene trees of each locus were obtained from the single locus BI (.t files generated by MrBayes), and were summarized by using the program mbsum [54] with a 25% burn-in. The output files of mbsum were then used to construct the primary concordance tree and concordance factor (CF) in the program bucky [54]. The *a priori* level of discordance among loci (α) was set to 0.01, 1 and 100 in alternative runs to assess the effect of α on the BCA results. Each run was conducted with 10,000,000 updates, four replicates, one cold and three hot chains of the Metropolis coupled Markov chain Monte Carlo (MCMCMC). In MCMCMC, a swap was proposed once every 100 updates, and the α multiplier was set to 10. Different runs with α set to 0.01, 1 and 100 all recovered the same primary concordance tree topology and CFs, hence the BCA results obtained with the default α = 1 were reported. The primary concordance tree was visualized in FigTree, and branch labels were annotated as the sample-wide posterior mean CFs and their 95% credibility intervals.

## Results

### Nuclear data

Characteristics of the individual nuclear datasets were summarized in Table 1. Treating indels as missing data or binary characters did not change the topologies, but the analyses that included indels as characters increased support values in general. The results presented here were based on the datasets with indels treated as binary characters. Trees generated from each individual datasets supported the monophyly of each of the genera *Ebenus* L., *Greuteria*, *Sulla*, and *Taverniera* DC., and several small lineages within *Hedysarum* and *Onobrychis*. *Hedysarum denticulatum* and *H*. *minjanense* were nested with *H*. sect. *Stracheya* as redefined by Duan et al. [11] in all five nuclear trees. Deep relationships within Hedysareae were largely unsolved by single sequences. However, *Hedysarum* was not supported to be monophyletic in any of the five single-sequence trees.

In order to search trees with better resolution, we concatenated individual datasets in our analyses. To explore whether two or more nuclear datasets could be concatenated, we conducted 10 pairwise ILD tests for the five nuclear datasets. The *p* values were shown in Table 2. Congruence was detected between any pair in the ETS, ITS, *PGDH* and *TRPT* datasets (all with *p* > 0.01). We first concatenated the non-coding ribosomal ETS and ITS datasets (pairwise ILD: *p* = 0.13), as well as the coding *PGDH* and *TRPT* datasets (pairwise ILD: *p* = 0.061) for two separate analyses.

**Table 2:** *P* values of pairwise ILD tests for the nuclear ETS, ITS, *PGDH*, *TRPT* and *SQD1* datasets.

	**ITS**	***PGDH***	***TRPT***	***SQD1***
**ETS**	0.130	0.036	0.025	0.001
**ITS**		0.331	0.170	0.002
***PGDH***			0.061	0.001
***TRPT***				0.003

In the concatenated ETS + ITS tree (S1 Fig), *Corethrodendron*, *Ebenus*, *Greuteria*, *Onobrychis*, *Sulla* and *Taverniera* were each supported to be monophyletic. *Corethrodendron*, *Eversmannia* Bunge, *Greuteria* and *Onobrychis* together formed a well supported clade (the CEGO clade), with Bayesian posterior probabilitiy (PP) = 1, maximum parsimony bootstrap percent (PBS) = 100%, and maximum likelihood bootstrap percent (LBS) = 100%, but relationships among these four genera were poorly resolved. The *Hedysarum* sect. *Hedysarum* clade and the *H*. sect. *Stracheya* clade were sisters (the *Hedysarum s*.*s*. clade, PP = 1, PBS = 70%, LBS = 94%). *Sartoria hedysaroides* plus some members of the *Hedysarum* sect. *Multicaulia s*.*l*. formed a well supported clade (the *Sartoria* clade, PP = 1, PBS = 100%, LBS = 100%). The genus *Hedysarum* was not monophyletic, because the *Hedysarum s*.*s*. clade grouped with the CEGO clade, while the *Sartoria* clade formed a clade with *Taverniera*. However, these relationships had low support values in the ITS + ETS tree (not supported by the BI tree, poorly supported by the MP and ML trees).

In the concatenated *PGDH* + *TRPT* tree (S2 Fig), the *Hedysarum* sect. *Hedysarum* clade and the *H*. sect. *Stracheya* clade were supported to be sister to each other and constituted the *Hedysarum s*.*s*. clade (PP = 0.98, PBS = 54%, LBS = 80%). Additionally, the *Hedysarum s*.*s*. clade grouped with *Corethrodendron*, *Eversmannia*, *Greuteria*, and *Onobrychis* (PP = 0.96, PBS = 34%, LBS = 35%), but relationships within this clade were unclear. The *Sartoria* clade (PP = 1, PBS = 71%, LBS = 76%) and *Taverniera* were supported to be sisters with low support (PP = 0.8, PBS = 52%, LBS = 64%).

In both the ETS + ITS tree (S1 Fig) and the *PGDH* + *TRPT* tree (S2 Fig), *Hedysarum* was not monophyletic because the *Hedysarum s*.*s*. clade grouped with *Corethrodendron*, *Eversmannia*, *Greuteria* and *Onobrychis*, while the *Sartoria* clade was sister to the genus *Taverniera*. Although support values varied in these two trees, *Hedysarum* was shown to be biphyletic. Pairwise ILD test between the ETS + ITS dataset and the *PGDH* + *TRPT* dataset found *p* = 0.703, thus we concatenated these four sequences in our further analyses.

In the concatenated ETS + ITS + *PGDH* + *TRPT* tree (S3 Fig), *Ebenus* diverged first, followed by *Sulla* (PP = 0.71, PBS = 84%, LBS = 40%). *Corethrodendron*, *Ebenus*, *Greuteria*, *Sulla*, and *Taverniera* were each highly supported to be monophyletic (all with PP = 1, PBS = 100%, LBS = 100%). *Onobrychis* was weakly supported to be monophyletic (PP = 0.68, PBS = 60%, not supported by the ML tree) with two strongly supported subclades (both with PP = 1, PBS = 100%, LBS = 100%). The CEGO clade was strongly supported (PP = 1, PBS = 100%, LBS = 100%). *Hedysarum* was biphyletic with the *Hedysarum s*.*s*. clade sister to the CEGO clade (PP = 1, PBS = 86%, LBS = 94%), and the *Sartoria* clade forming a clade with *Taverniera* (PP = 0.97, PBS = 69%, LBS = 92%). The CEGO + *Hedysarum s*.*s*. clade was then sister to the *Sartoria* + *Taverniera* clade (PP = 1, PBS = 82%, LBS = 90%).

The *SQD1* tree (S4 Fig) showed limited resolution as well as low support values concerning the deep relationship in Hedysareae. The *SQD1* dataset has the smallest number (41) of parsimony-informative sites compared with the other four nuclear sequences (see Table 1). *Hedysarum* was, nevertheless, not monophyletic in the *SQD1* tree. Members of the *Sartoria* clade grouped with *Taverniera*, *Hedysarum longigynophorum* C.C.Ni, *H*. *dentatoalarum* K.T.Fu and *H*. *chinense* (B.Fedtsch.) Hand.-Mazz., but the support values were low (PP = 0.85, PBS = 52%, LBS = 53%). *Sulla*, *Ebenus*, *Eversmannia*, *Corethrodendron*, *Greuteria*, *Onobrychis* and the rest of *Hedysarum* formed another clade (PP = 1, PBS = 62%, LBS = 68%). We could see that the *SQD1* tree (S4 Fig) was not contrary to the ETS + ITS + *PGDH* + *TRPT* tree (S3 Fig) with regard to the non-monophyly of *Hedysarum*. Hence we tentatively concatenated the *SQD1* dataset with the other four nuclear datasets.

**Fig 1:**
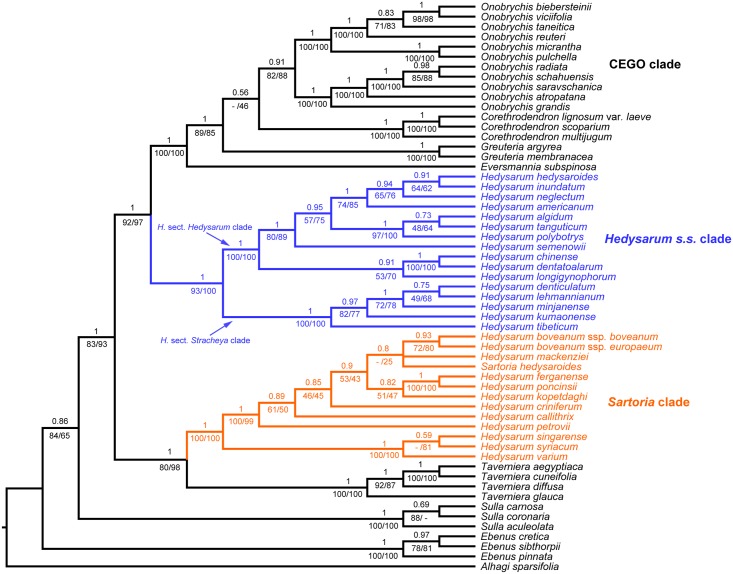
Bayesian tree based on the concatenated nuclear ITS, ETS, *PGDH*, *TRPT* and *SQD1* sequences. The Bayesian posterior probabilities are above the branches, and the maximum parsimony (left) and maximum likelihood (right) bootstrap support values are below the branches. Dashes indicate branches that are not found in the maximum parsimony tree or the maximum likelihood tree.

In the concatenated ETS + ITS + *PGDH* + *TRPT* + *SQD1* tree (Fig 1), relationships of the CEGO, *Hedysarum s*.*s*., *Sartoria* clades and *Taverniera* remained the same as in the concatenated ETS + ITS + *PGDH* + *TRPT* tree (S3 Fig). With the *SQD1* dataset added, the support values were even higher (Fig 1): the *Hedysarum s*.*s*. clade was sister to the CEGO clade with PP = 1, PBS = 92%, LBS = 97% (compared with PP = 1, PBS = 86%, LBS = 94% in S3 Fig), and the *Sartoria* clade grouped with *Taverniera* with PP = 1, PBS = 80%, LBS = 98% (compared with PP = 0.97, PBS = 69%, LBS = 92% in S3 Fig).

The coalescent tree based on all five nuclear sequences (S5 Fig) revealed that the *Hedysarum s*.*s* clade was sister to the CEGO clade (PP = 0.95), and the *Sartoria* clade grouped with *Taverniera* (PP = 0.96). The CEGO, *Hedysarum s*.*s*., the *Sartoria* clade and *Taverniera* were each supported to be monophyletic (all with PP = 1).

The primary concordance tree based on all five nuclear sequences (S6 Fig) had similar topology compared with the concatenated data tree (Fig 1) and the coalescent tree (S5 Fig). It supported the sister relationship between the *Hedysarum s*.*s* and the CEGO clades (mean CF = 0.26), and that between the *Sartoria* clade and *Taverniera* (mean CF = 0.3).

### Plastid data

Characteristics of plastid datasets were presented in Table 1. Trees based on individual plastid datasets had limited resolution. The 10 pairwise ILD tests for the five plastid datasets all found *p* > 0.01 (Table 3), therefore we concatenated these five plastid datasets in our analyses. Because there was incongruence between the plastid and nuclear trees, the plastid and nuclear datasets were not concatenated in our analysis.

**Table 3:** *P* values of pairwise ILD tests for the plastid *psbA-trnH*, *trnC-petN*, *trnL-trnF*, *trnS-trnG* and *petN-psbM* datasets.

	***trnC-petN***	***trnL-trnF***	***trnS-trnG***	***petN-psbM***
***psbA-trnH***	0.751	0.266	0.423	0.315
***trnC-petN***		0.143	0.192	0.894
***trnL-trnF***			0.260	0.657
***trnS-trnG***				0.250

**Fig 2:**
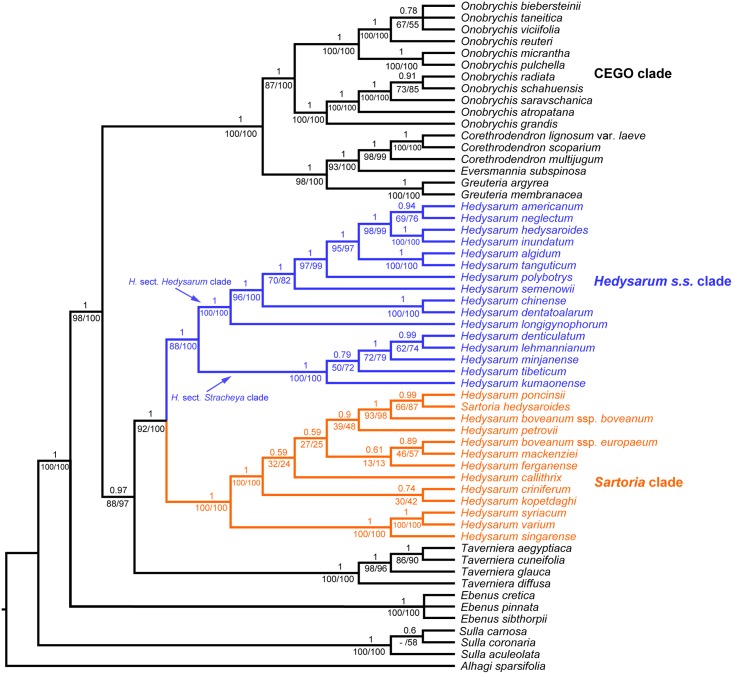
Bayesian tree based on the concatenated plastid *psbA-trnH*, *trnC-petN*, *trnL-trnF*, *trnS-trnG*, and *petN-psbM* sequences. The Bayesian posterior probabilities are above the branches, and the maximum parsimony (left) and maximum likelihood (right) bootstrap support values are below the branches. A dash indicates a branch that is not found in the maximum parsimony tree.

In the concatenated plastid tree based on *psbA-trnH*, *trnC-petN*, *trnL-trnF*, *trnS-trnG* and *petN-psbM* sequences (Fig 2), *Sulla* diverged first, followed by *Ebenus* with high support (PP = 1, PBS = 100%, LBS = 100%). *Corethrodendron*, *Ebenus*, *Greuteria*, *Hedysarum*, *Onobrychis*, *Sulla*, and *Taverniera* were each supported to be monophyletic. The CEGO clade was also highly supported (PP = 1, PBS = 100%, LBS = 100%), which was sister to the *Hedysarum + Taverniera* clade (PP = 1, PBS = 98%, LBS = 100%). *Hedysarum* was monophyletic (PP = 1, PBS = 92%, LBS = 100%), and was sister to *Taverniera* (PP = 0.97, PBS = 88%, LBS = 97%). Within *Hedysarum*, the *Sartoria* clade (PP = 1, PBS = 100%, LBS = 100%) and the *Hedysarum s*.*s*. clade (PP = 1, PBS = 88%, LBS = 100%) were each well supported. *H*. *denticulatum* and *H*. *minjanense* were nested within *H*. sect. *Stracheya* as redefined by Duan et al. [11]. The *Hedysarum* sect. *Hedysarum* clade was sister to the *H*. sect. *Stracheya* clade (PP = 1, PBS = 88%, LBS = 100%).

The coalescent tree based on all five plastid sequences (S7 Fig) showed that *Hedysarum* was monophyletic (PP = 1), and was sister to *Taverniera* (PP = 0.83). The *Hedysarum* + *Taverniera* clade was then sister to the CEGO clade (PP = 1).

The primary concordance tree based on all five plastid sequences (S8 Fig) also had similar topology compared with the concatenated plastid data tree (Fig 2) and the coalescent tree (S7 Fig). *Hedysarum* was supported to be monophyletic (mean CF = 0.67) and was sister to *Taverniera* (mean CF = 0.35). The *Hedysarum* + *Taverniera* clade was then sister to the CEGO clade (mean CF = 0.56).

The AU tests of the concatenated nuclear ETS + ITS + *PGDH* + *TRPT* dataset against the concatenated five plastid sequence dataset were performed. The nuclear dataset rejected the plastid topology (*p* < 0.01), and the plastid dataset rejected the nuclear topology (*p* < 0.01) as well, suggesting incongruence between the nuclear and plastid datasets. The concatenated five nuclear sequence dataset (ETS + ITS + *PGDH* + *TRPT + SQD1*) also rejected the plastid topology (*p* < 0.01), and vice versa.

## Discussion

### Non-monophyly of *Hedysarum*

**Fig 3:**
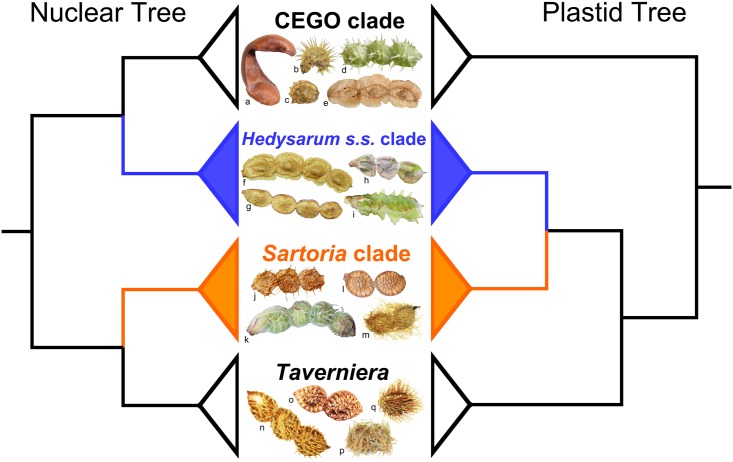
Summary of the incongruent position of the *Hedysarum s*.*s*. clade in the nuclear (left) and the plastid (right) trees, with selected legume species from each clade (middle). The legumes: a, *Eversmannia subspinosa* B.Fedtsch.; b, *Onobrychis atropatana* Boiss.; c, *O*. *viciifolia* Scop.; d, *Corethrodendron multijugum* (Maxim.) B.H.Choi & H.Ohashi; e, *Greuteria membranacea* (Coss. & Balansa) Amirahm. & Kaz.Osaloo; f, *Hedysarum semenowii* Regel & Herder; g, *H*. *americanum* (Michx.) Britton; h, *H*. *kumaonense* Benth. ex Baker; i, *H*. *tibeticum* (Benth.) B.H.Choi & H.Ohashi; j, *H*. *gremiale* Rollins; k, *H*. *petrovii* Yakovlev; l, *H*. *boveanum* Bunge ex Basiner; m, *H*. *callithrix* Bunge ex Boiss.; n, *Taverniera diffusa* (Cambess.) Thulin; o, *T*. *glauca* Edgew.; p, *T*. *lappacea* DC.; q, *T*. *longisetosa* Thulin; photoed by PLL and LD.

Our results revealed incongruence between the nuclear and the plastid trees in *Hedysarum* (Fig 3). In the nuclear trees (Figs 1 and 3), *Hedysarum* is biphyletic; the *Sartoria* clade is sister to *Taverniera*; and the *Hedysarum s*.*s*. clade is sister to the CEGO clade. On the other hand, *Hedysarum* is monophyletic and sister to *Taverniera* in the plastid tree (Figs 2 and 3). The topological incongruence concerning *Hedysarum* is robustly supported (Figs 1 and 2). The difference between the nuclear and the plastid topologies lies on the position of the *Hedysarum* s.s. clade, with the *Hedysarum* s.s. clade sister to the CEGO clade in the nuclear tree, in contrast with its sister relationship with the *Sartoria* clade in the plastid tree (Fig 3).

Our trees based on concatenated nuclear and plastid data are well supported concerning the position of the *Hedysarum s*.*s*. clade (Figs 1 and 2). However, there is concern that data concatenation may lead to overconfidence in incorrect species tree [55]. Thus we also used the coalescent method and BCA to infer the phylogenetic relationships using multi-locus data. Our coalescent trees (S5 and S7 Figs) agree with the concatenated trees (Figs 1 and 2), and they are also well supported. In BCA, the concordance factor (CF) of a certain clade is the proportion of the genes or the genome for which that given clade is true, and should not be confused with a clade support value [53, 56]. In our BCA results (S6 and S8 Figs), CFs of many clades are below 1, which is likely due to the limited number of phylogenetic informative sites in each individual locus especially in the *PGDH*, *SQD1*, *TRPT* and *psbA-trnH* sequences (Table 1). The single locus trees show limited resolution with many polytomies, such as the *SQD1* tree (S4 Fig). The nuclear sequences have fewer informative sites than the plastid ones (Table 1), and the CFs from the nuclear BCA are lower than those from the plastid BCA (S6 and S8 Figs). However, in the nuclear as well as in the plastid BCA results, the primary concordance tree is built from the clades with highest CFs and shows the relationships shared by a large proportion of the sampled genes [56]. It agrees with the concatenated data tree and the multi-locus coalescent tree. Closer inspection of the nuclear BCA result shows that in the list of clades which are not in the nuclear primary concordance tree but with estimated CF > 0.05, there is no such clade of a monophyletic *Hedysarum* as in the plastid phylogeny. Also, in the list of clades which are not in the plastid primary concordance tree but with estimated CF > 0.05, there is no contradictory clade of a biphyletic *Hedysarum* as in the nuclear phylogeny. In our analyses, various methods of phylogenetic reconstructions, i.e., BI, MP and ML based on concatenated data, multi-locus coalescent, and BCA, all uncover the same incongruent pattern between the nuclear and the plastid trees (Fig 3).

Various mechanisms have been proposed to explain topological incongruence between different gene trees, such as hybridization/introgression, lineage sorting, allopolyploidy, horizontal gene transfer, and many other factors [14, 57–59].

Insufficient data is probably responsible for the low resolution of the *SQD1* tree, however it can be ruled out from the candidate mechanisms that explain the incongruent position of the *Hedysarum s*.*s*. clade because both the nuclear and plastid topologies are robustly supported (Figs 1 and 2, S3 Fig). Gene choice and rate of molecular evolution [58] can also be ruled out for the same reason. Sequencing error probably affects phylogenetic accuracy where there are a small number of informative sites [58]. In our trees, sequencing error at most only influences the closely related terminals where the support is low, but should not affect well supported deep relationships. Our sampling covers all genera of the tribe Hedysareae and all sections and major infra-sectional clades in *Hedysarum* as recognized by Duan et al. [11]. Furthermore the different sequences were generated from the same DNA extraction (except for the *PGDH* sequence of *Sulla coronaria*, see S1 Appendix) for each accession. Insufficient taxon sampling and sample misidentification are thus not likely to be the causes of the deep incongruence.

Incomplete homogenization of tandem repeats and the possibly uniparental inheritance of the nuclear ribosomal gene region, and orthology/paralogy conflation of the nuclear low or single copy genes, may affect the accuracy of phylogenetic reconstruction [13, 58, 60, 61]. These issues may lead to incongruence between different nuclear gene trees. We cannot fully rule out these issues in our nuclear data. However, the results based on ribosomal gene regions (S1 Fig) and low or single copy genes (S2 and S4 Figs) all support a biphyletic *Hedysarum*. Furthermore, the monophyly of *Hedysarum* is not detected in any of the nuclear trees. Therefore, the incongruence between the nuclear and the plastid trees cannot be simply explained by such mechanisms.

Allopolyploidy can be excluded from being considered as a mechanism to explain the incongruent position of the *Hedysarum s*.*s*. clade. We checked the chromosome number counts for Hedysareae in the *Index to Plant Chromosome Numbers* (IPCN, http://www.tropicos.org/Project/IPCN). Most species in *Hedysarum* as well as those of other genera in Hedysareae have been reported to have chromosome numbers 2*n* = 16 or 2*n* = 14, suggesting that they are diploid because their basic chromosome numbers are indicated as *x* = 8 or *x* = 7 [2, 62]. To summarize the available data from IPCN, deep lineages of Hedysareae are predominantly diploid, e.g., 2*n* = 2*x* = 16 for *Alhagi*, *Sulla* (species name still under *Hedysarum* in IPCN; with one report of 2*n* = 18), *Taverniera*, *Corethrodendron* (species name still under *Hedysarum* in IPCN), *Eversmannia*, the *Hedysarum* sect. *Stracheya* clade (*H*. *minjanense* and *H*. *denticulatum*), and 2*n* = 2*x* = 14 for *Ebenus* (with one report of 2*n* = 18). Most species in the *Sartoria* clade, the *H*. sect. *Hedysarum* clade and *Onobrychis* are diploid with chromosome numbers 2*n* = 14 or 16. Polyploidy in *Hedysarum* and *Onobrychis* occurs mostly as multiple cytotypes within one species (e.g., 2*n* = 14, 28 for *H*. *hedysaroides* Schinz & Thell., 2*n* = 16, 32 for *H*. *mackenziei* Richardson), with some being exclusively polyploid such as 2*n* = 28 for *H*. *inundatum* Turcz. and *O*. *biebersteinii* Širj. [63].

Occasionally horizontal gene transfer between species through nonsexual means may explain gene tree incongruence [58]. But the variational pattern in our study would require multiple horizontal transfers in the nuclear genes (ETS, ITS, *PGDH*, *TRPT*, *SQD1*) in a particular ancestor of *Hedysarum*, which simultaneously leads to discordant gene trees with the plastid tree. This hypothesis is viewed difficult, as many unlinked genes are not likely to be horizontally transferred in parallel [64]. Stegemann et al. [65] showed that an entire chloroplast genome could be horizontally transferred between sexually incompatible species through grafting. Their results were based on artificial grafting and selection process. It is not known whether chloroplast genome could be horizontally transferred through natural grafting. Additionally, unlike in woody plants, species of Hedysareae are not likely to form natural grafting because most species are herbaceous perennials.

Lineage sorting of ancestral polymorphisms can cause incongruent gene trees. The phylogenetic consequence of lineage sorting is usually difficult to distinguish from that of the other processes, such as hybridization/introgression [58, 66]. In general, lineage sorting is expected to be a possible cause of incongruence at lower taxonomic ranks often in population and/or species-level studies [58]. But the incongruence in our results involves deep relationships among different genera. Lineage sorting may contribute to incongruence at higher taxonomic ranks when polymorphisms are maintained by balancing selection through many speciation events [58]. Concerning the position of the *Hedysarum s*.*s*. clade, however, our tree based on coding genes (*PGDH*, *TRPT*) agrees with the tree based on non-coding sequences (ETS, ITS), which are thought to be neutral. Our tree based on the plastid non-coding data (Fig 2) also agrees with the tree based on the plastid coding *matK* gene in a previous study [10]. Hence lineage sorting may not be the best explanation for the incongruence in our results.

Our phylogenetic pattern may be best explained by a chloroplast capture hypothesis via introgression. In most angiosperm taxa, the plastid genome is uniparentally inherited, whereas the nuclear genome is biparentally inherited [13, 67]. After many generations of backcrossing through introgression, the introgressant inherited the plastid genome from one parent, and nearly all nuclear genes from the other parent. A significant feature of chloroplast capture is that plastid genome introgression is always not accompanied by nuclear introgression [14, 58, 65]. Our results are consistent with this scenario, i.e., multiple nuclear datasets are not supporting the monophyletic *Hedysarum* as suggested by the plastid tree. Chloroplast capture is probably one of the most common causes of phylogenetic incongruence [58], and has been reported in a wide range of taxa at various taxonomic levels [14, 15, 68–75].

Our phylogenetic analyses of the nuclear sequences show that *Hedysarum* is not monophyletic. Its generic delimitation needs to be reconsidered. The *Sartoria* clade and the *Hedysarum s*.*s*. clade can be distinguished from each other by several morphological characters. Most species in the *Sartoria* clade have grayish-green leaves, obscure lateral veins in the leaflets, biconvex and non-winged loments which always possess prickles, bristles, and ribs, whereas species in the *Hedysarum s*.*s*. clade always have bright-green leaves, visible lateral veins in the leaflets, compressed and more or less winged loments which are always unarmed [2, 3, 5]. Loment morphology of the *Sartoria* clade is very similar to that of *Taverniera* by its prickles, bristles, and ribs [2, 76], corresponding to the sister relationship between the *Sartoria* clade and *Taverniera* in the nuclear tree (Fig 3). The compressed, unarmed, more or less winged loment is also found in *Greuteria* and *Eversmannia* in the CEGO clade (Fig 3) [10]. Mironov [77] found that species of *H*. sect. *Gamotion* (members of the *Hedysarum s*.*s*. clade) differed from species of *H*. sect. *Multicaulia* (members of the *Sartoria* clade) in several pericarp anatomical characters that were thought to be of systematic significance.

Additionally, the *Hedysarum s*.*s*. clade and the *Sartoria* clade also show eco-geographical differentiation. In general, members of the *Hedysarum s*.*s*. clade adapt to mesic and/or psychric habitats in temperate montane forests, alpine and arctic regions of Eurasia and North America, whereas species of the *Sartoria* clade are distributed in xeric habitats in arid/semi-arid areas and steppes of central and western Asia, the Mediterranean region and western North America [11, 78].

### Systematic position of the *Hedysarum* sect. *Stracheya* clade

*Stracheya* was originally established as a monotypic genus [9], which was then treated as a section of *Hedysarum* based on comprehensive morphological analyses [2, 3]. *Hedysarum tibeticum* was the sole member of *H*. sect. *Stracheya* when Choi and Ohashi first constructed this section in *Hedysarum* [2]. However, previous phylogenetic analyses showed different systematic positions of the *H*. sect. *Stracheya* clade in Hedysareae with low support values [10, 11]. With more sequence data, our nuclear and plastid trees resolved congruent sister relationship between the *H*. sect. *Hedysarum* clade and a clade of five species including *H*. *tibeticum*, *H*. *kumaonense*, *H*. *lehmannianum*, *H*. *denticulatum*, and *H*. *minjanense* (Figs 1 and 2, S3 Fig). Our analysis supports merging *Stracheya* with *Hedysarum* and expanding *H*. sect. *Stracheya* to include four additional species. These four species were previously treated as members of *H*. sect. *Subacaulia* because of their strongly reduced stems [5, 20]. However, they did not form a clade with other members of *H*. sect. *Subacaulia* such as *H*. *petrovii*, *H*. *ferganense* Korsh., and *H*. *poncinsii* (Figs 1 and 2, S3 Fig). Some species of *H*. sect. *Hedysarum*, for example, *H*. *tanguticum* B.Fedtsch. and *H*. *pseudoastragalus* Ulbr., also have reduced stems [5]. Thus, the strongly reduced stem is most likely a homoplasious convergence in *Hedysarum*. The dwarf habit of the *H*. sect. *Stracheya* lineage might be an adaptation to the cold and arid habitats in the pan-Himalayan region and the adjacent eastern part of central Asia [79, 80].

## Supporting Information

S1 Appendix Taxon names, geographical locality, voucher information and herbarium codes, and GenBank accession numbers for the sequences used in this study. Herbarium codes follow the Index Herbariorum (http://sweetgum.nybg.org/ih/). For each taxon, GenBank accession numbers of the ten sequences are given in the sequence of ETS, ITS, *PGDH*, *SQD1*, *TRPT*, *trnL-trnF*, *psbA-trnH*, *trnS-trnG*, *trnC-petN*, and *petN-psbM*. New sequences generated in this study are indicated by an asterisk (*). Missing sequences are indicated by a dash (-).
(XLS)

S1 Fig Maximum parsimony tree based on the concatenated nuclear ITS and ETS sequences. The Bayesian posterior probabilities are above the branches, and the maximum parsimony (left) and maximum likelihood (right) bootstrap support values are below the branches. Dashes indicate branches that are not found in the Bayesian tree or the maximum likelihood tree.
(TIF)

S2 Fig Bayesian tree based on the concatenated nuclear *PGDH* and *TRPT* sequences. The Bayesian posterior probabilities are above the branches, and the maximum parsimony (left) and maximum likelihood (right) bootstrap support values are below the branches. Dashes indicate branches that are not found in the maximum parsimony tree or the maximum likelihood tree.
(TIF)

S3 Fig Bayesian tree based on the concatenated nuclear ITS, ETS, *PGDH* and *TRPT* sequences. The Bayesian posterior probabilities are above the branches, and the maximum parsimony (left) and maximum likelihood (right) bootstrap support values are below the branches. Dashes indicate branches that are not found in the maximum parsimony or the maximum likelihood trees.
(TIF)

S4 Fig Bayesian tree based on nuclear *SQD1* sequences. The Bayesian posterior probabilities are above the branches, and the maximum parsimony (left) and maximum likelihood (right) bootstrap support values are below the branches. Dashes indicate branches that are not found in the maximum parsimony or the maximum likelihood trees.
(TIF)

S5 Fig Coalescent tree based on nuclear ETS, ITS, *PGDH*, *SQD1* and *TRPT* sequences. The posterior probabilities are above the branches.
(TIF)

S6 Fig Primary concordance tree based on nuclear ETS, ITS, *PGDH*, *SQD1* and *TRPT* sequences. The sample-wide posterior mean concordance factors are above the branches, and their 95% credibility intervals are below the branches.
(TIF)

S7 Fig Coalescent tree based on plastid *psbA-trnH*, *trnC-petN*, *trnL-trnF*, *trnS-trnG* and *petN-psbM* sequences. The posterior probabilities are above the branches.
(TIF)

S8 Fig Primary concordance tree based on plastid *psbA-trnH*, *trnC-petN*, *trnL-trnF*, *trnS-trnG* and *petN-psbM* sequences. The sample-wide posterior mean concordance factors are above the branches, and their 95% credibility intervals are below the branches.
(TIF)
