# Supplementary figures and images for: Hedysarum L. (Fabaceae: Hedysareae) Is Not Monophyletic – Evidence from Phylogenetic Analyses Based on Five Nuclear and Five Plastid Sequences
Source: PLoS One. 2017 Jan 25;12(1):e0170596. doi: 10.1371/journal.pone.0170596 (PMC5266277; doi:10.1371/journal.pone.0170596)

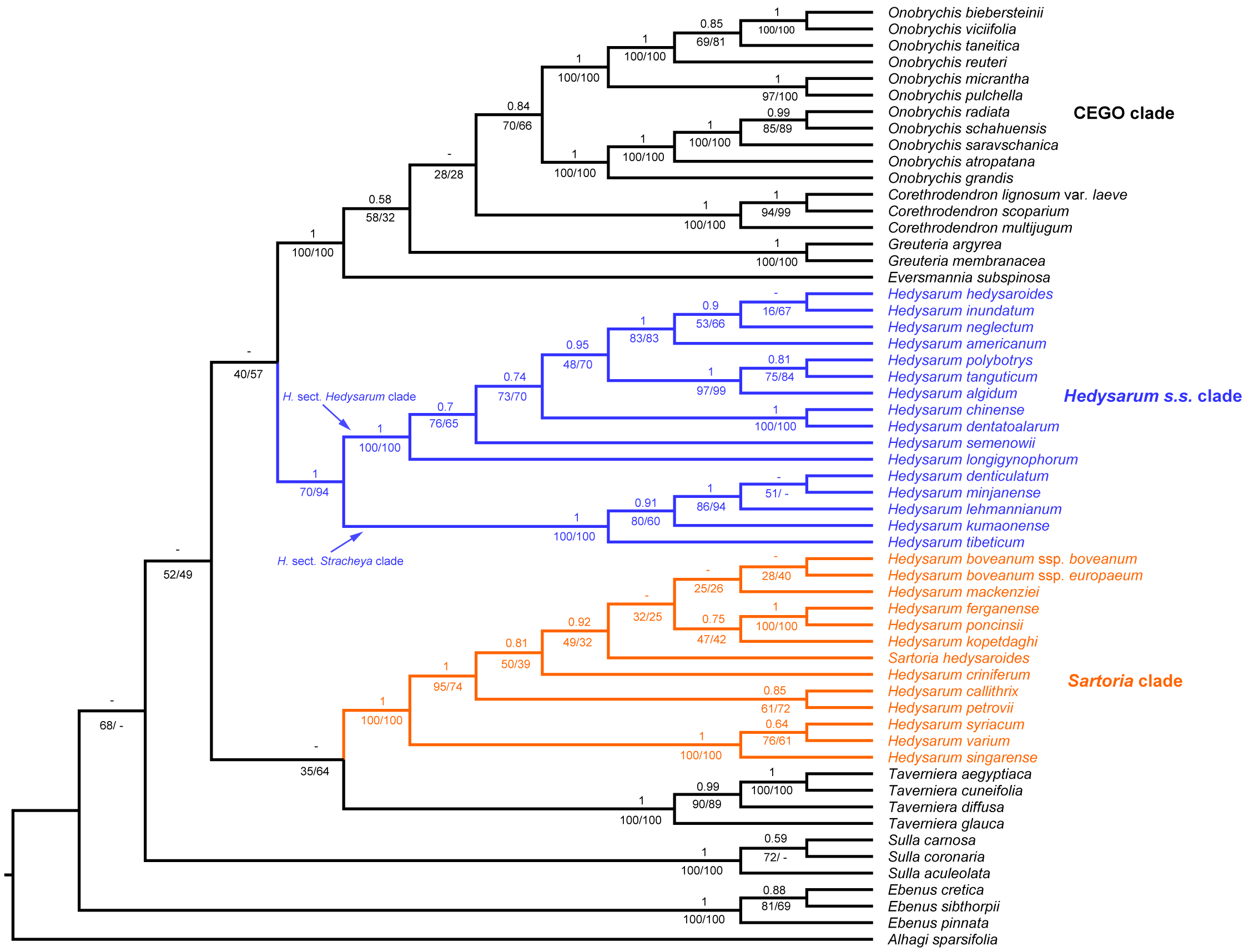

Supplement: S1 Fig — The Bayesian posterior probabilities are above the branches, and the maximum parsimony (left) and maximum likelihood (right) bootstrap support values are below the branches. Dashes indicate branches that are not found in the Bayesian tree or the maximum likelihood tree. (TIF) [file pone.0170596.s002.tif]

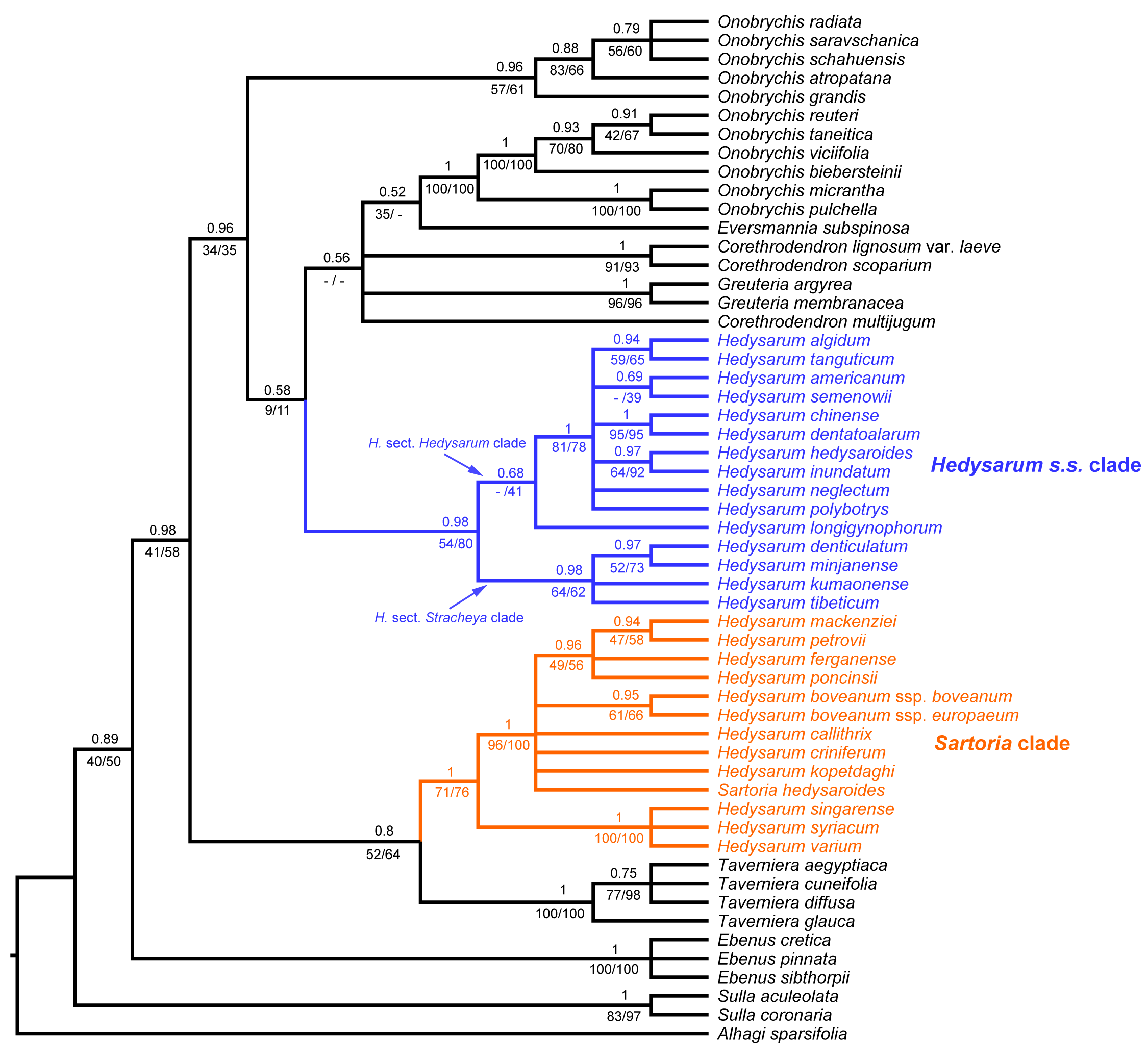

Supplement: S2 Fig — The Bayesian posterior probabilities are above the branches, and the maximum parsimony (left) and maximum likelihood (right) bootstrap support values are below the branches. Dashes indicate branches that are not found in the maximum parsimony tree or the maximum likelihood tree. (TIF) [file pone.0170596.s003.tif]

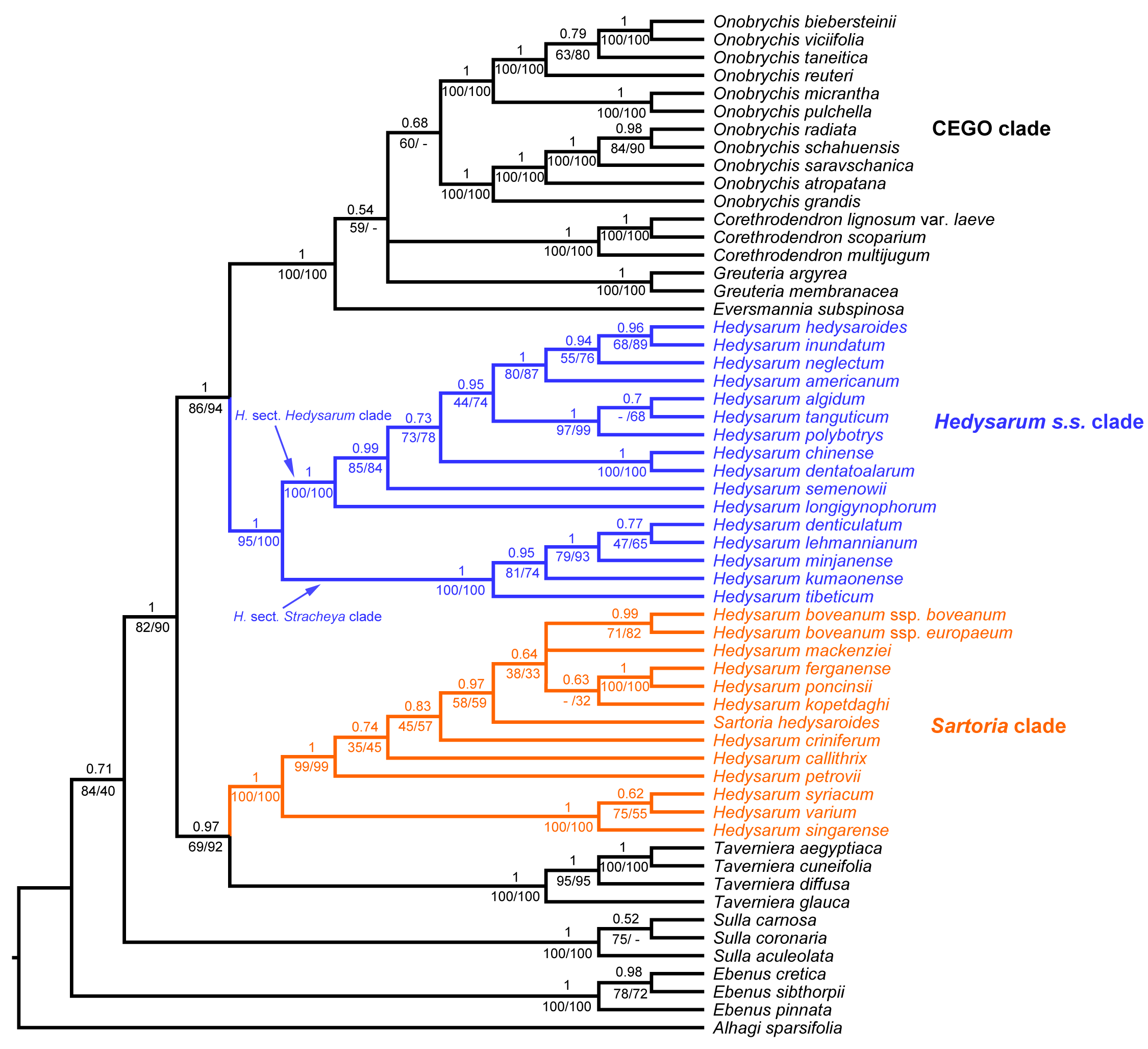

Supplement: S3 Fig — The Bayesian posterior probabilities are above the branches, and the maximum parsimony (left) and maximum likelihood (right) bootstrap support values are below the branches. Dashes indicate branches that are not found in the maximum parsimony or the maximum likelihood trees. (TIF) [file pone.0170596.s004.tif]

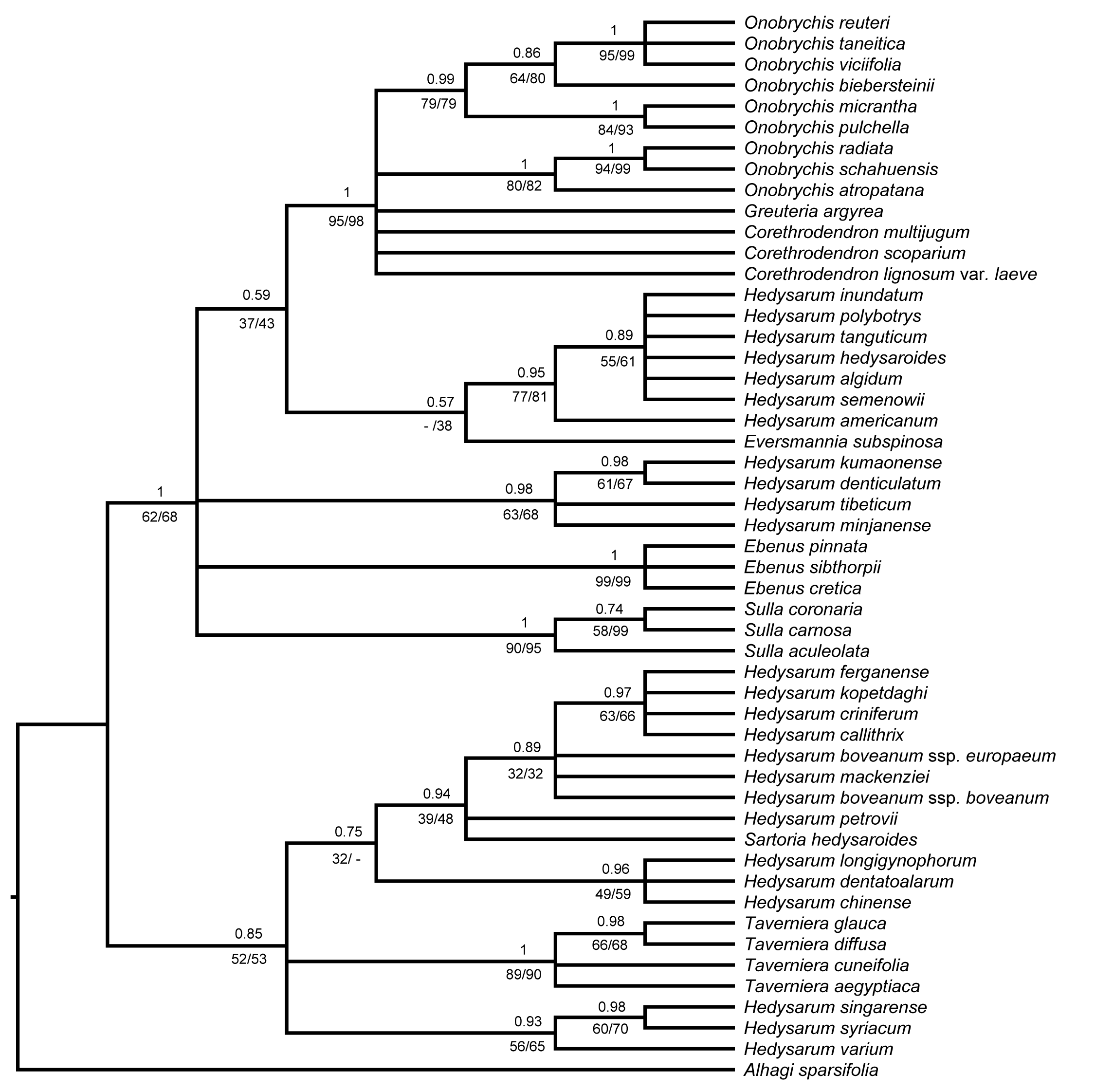

Supplement: S4 Fig — The Bayesian posterior probabilities are above the branches, and the maximum parsimony (left) and maximum likelihood (right) bootstrap support values are below the branches. Dashes indicate branches that are not found in the maximum parsimony or the maximum likelihood trees. (TIF) [file pone.0170596.s005.tif]

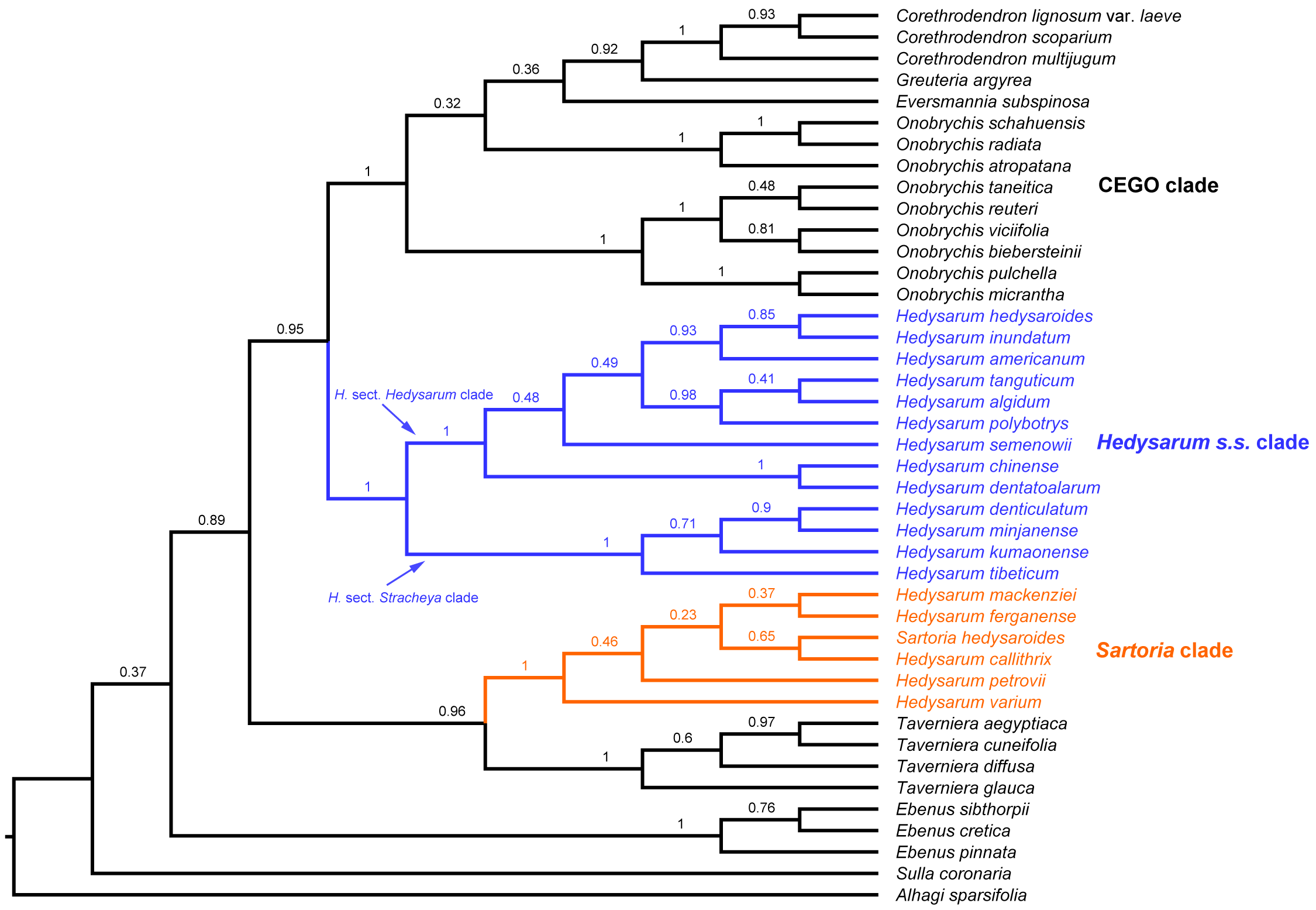

Supplement: S5 Fig — The posterior probabilities are above the branches. (TIF) [file pone.0170596.s006.tif]

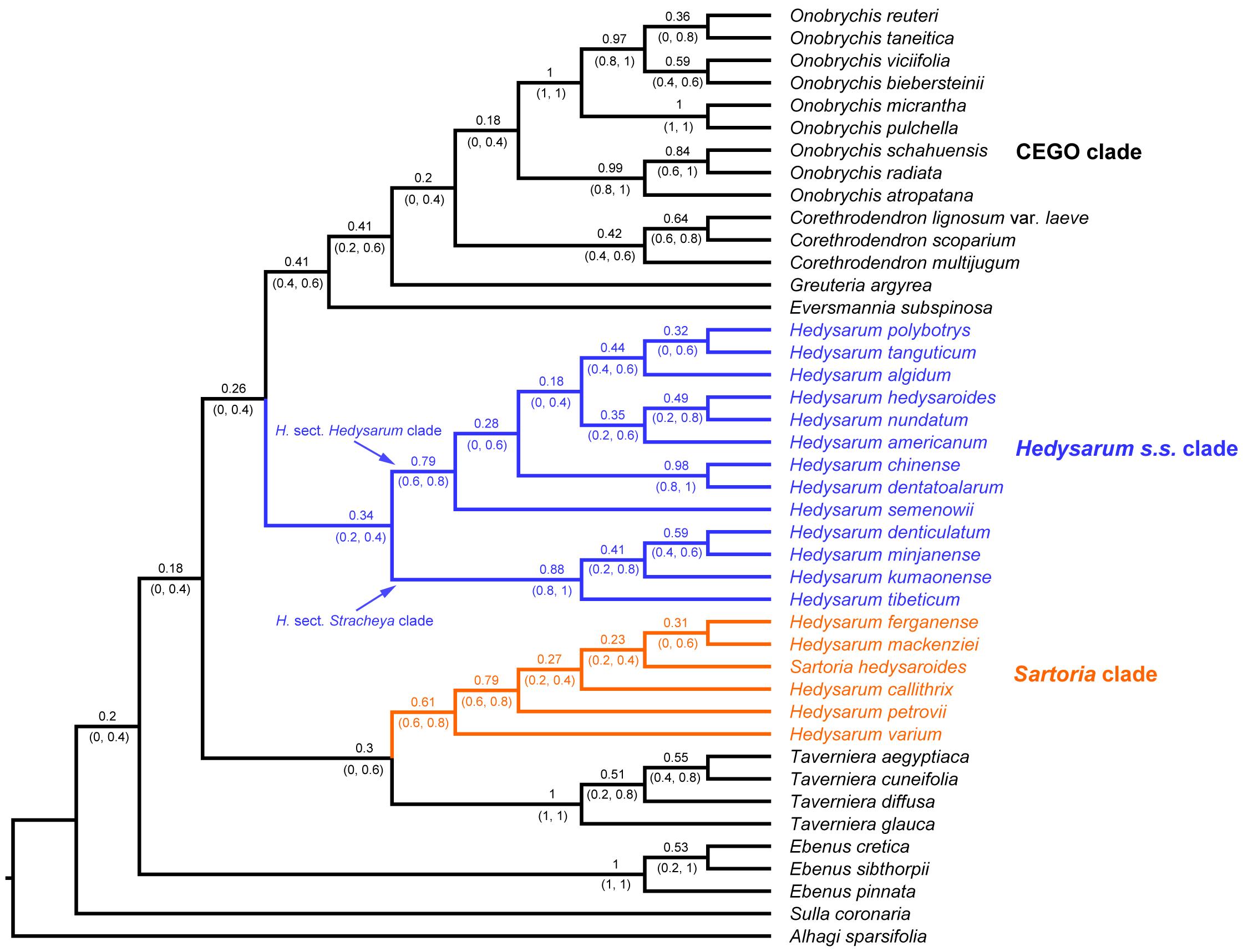

Supplement: S6 Fig — The sample-wide posterior mean concordance factors are above the branches, and their 95% credibility intervals are below the branches. (TIF) [file pone.0170596.s007.tif]

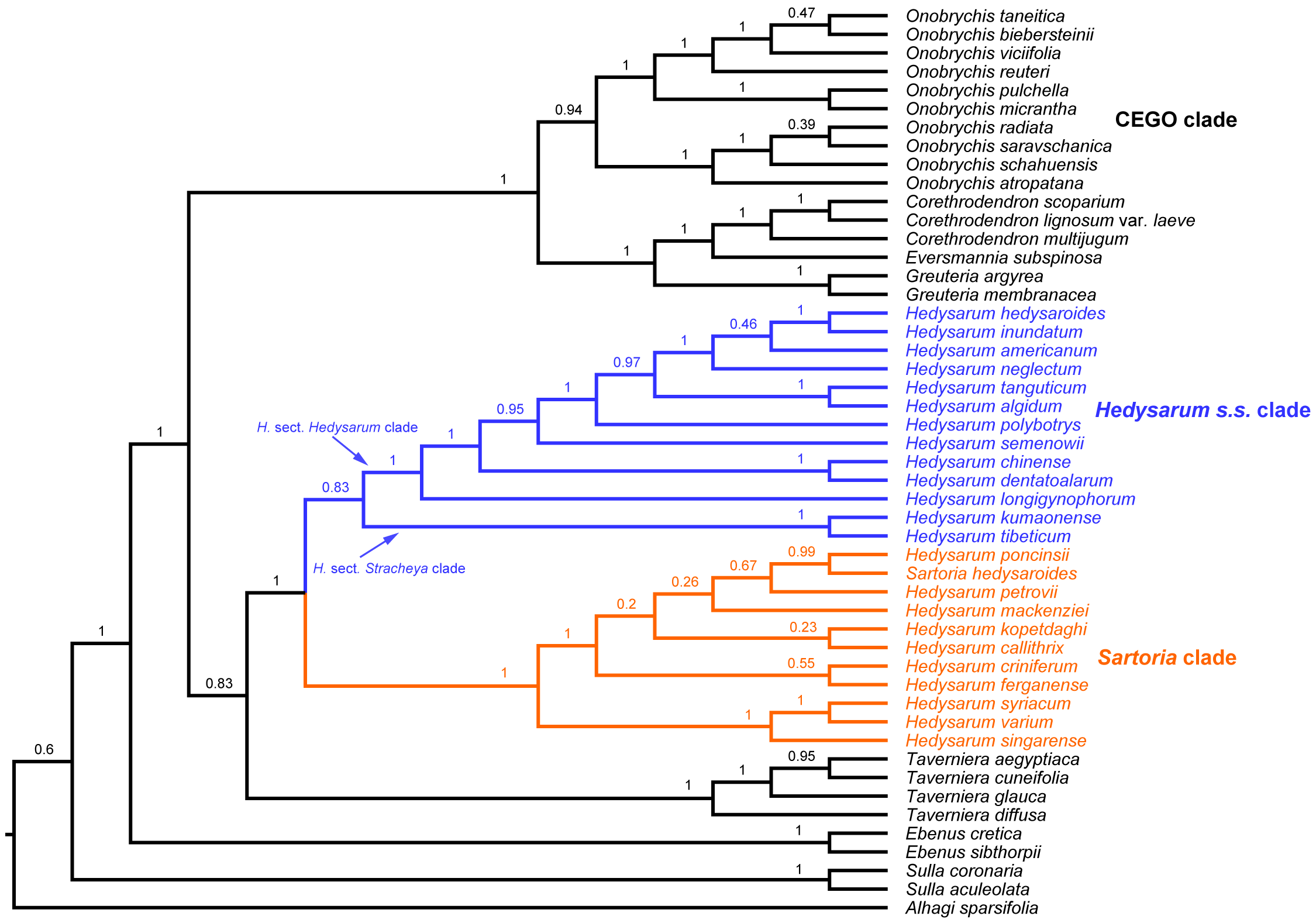

Supplement: S7 Fig — The posterior probabilities are above the branches. (TIF) [file pone.0170596.s008.tif]

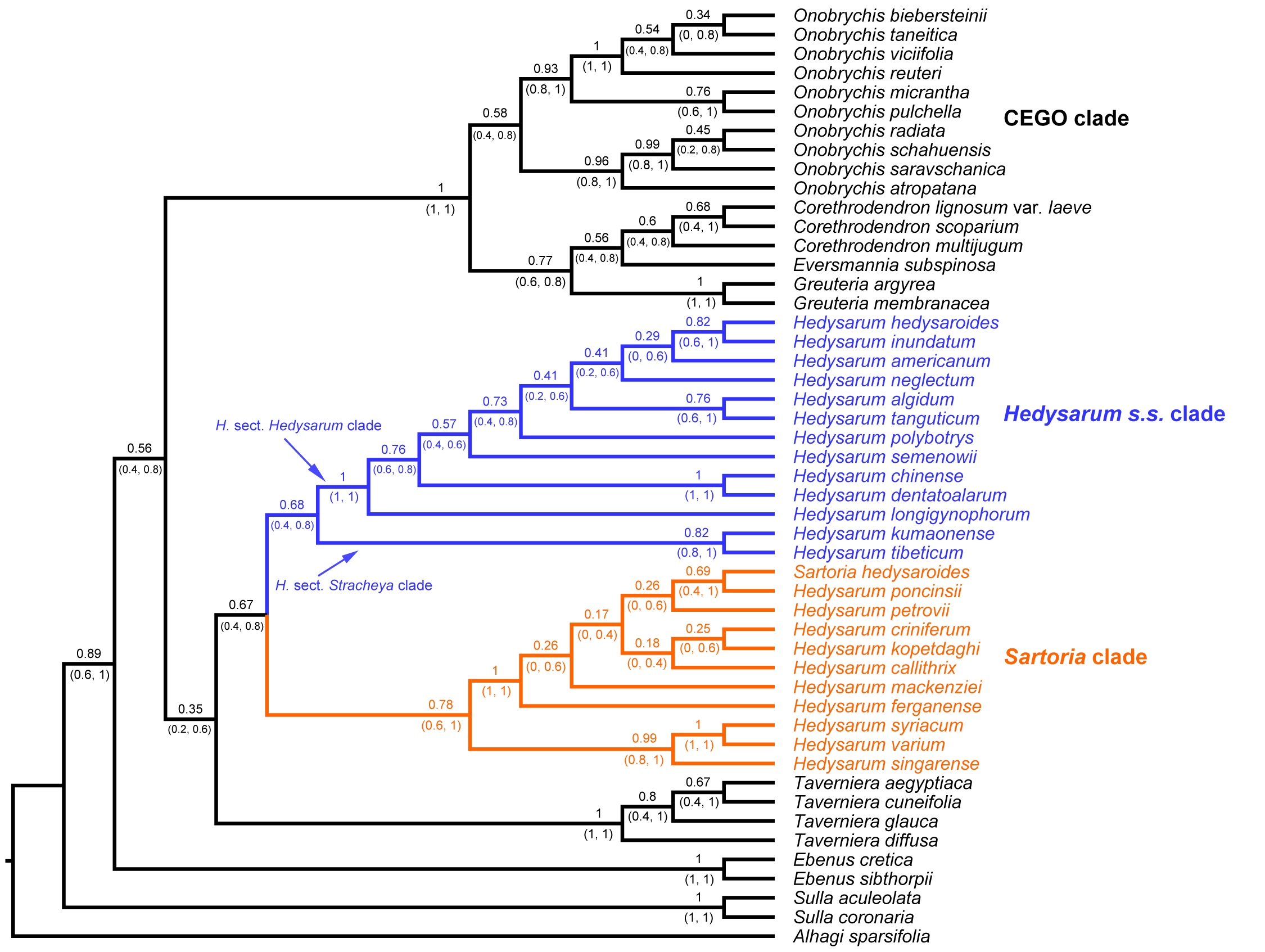

Supplement: S8 Fig — The sample-wide posterior mean concordance factors are above the branches, and their 95% credibility intervals are below the branches. (TIF) [file pone.0170596.s009.tif]
